# Supplementary material for: A neural network-based model framework for cell-fate decisions and development
Source: Commun Biol. 2024 Mar 14;7:323. doi: 10.1038/s42003-024-05985-1 (PMC10940658; doi:10.1038/s42003-024-05985-1)
Supplement: Supplementary file 2 — Supplementary Material [file 42003_2024_5985_MOESM2_ESM.pdf]

Supplementary Information for

A neural network-based model framework for cell-fate decisions and development

Mátyás Paczkó, Dániel Vörös, Péter Szabó, Gáspár Jékely, Eörs Szathmáry, András Szilágyi

Table of contents

Supplementary Note 1. Step-wise regulatory program matrix derivation 2

Supplementary Note 2. Landscape mapping 4

Supplementary Note 3. Effect of the degradation rate 5

Supplementary Note 4. Regulatory network modularity..6

Supplementary Note 5. An organogenetic AGRN model 7

Supplementary Note 6. Subcircuits 9

Supplementary References 11

Supplementary Note 1. Step-wise regulatory program matrix derivation

In order to demonstrate the derivation of a regulatory program matrix in a manner that it can be assembled for an arbitrary developmental system by applying the rules of the three elementary transition types of the AGRN model (Eqs. 4-6, Fig. 1b, Fig. 8a), here we give a detailed description of the step-wise building-up process of the **M** regulatory program matrix governing the developmental system illustrated in Fig. 8b. The structure of a developmental stage vector used for matrix construction is as follows in this example: 1.-6. vector elements represent expressions of stage-specific genes in the 6 stages (A-F) of the system. Due to the fact that 'minimal expression representation' is considered, exactly one gene, which is unique for a certain stage, is expressed in each stage. The 7th vector element is the trigger for the fork transition, while the 8th vector element is the trigger for the conditional transition. The stage vectors are:

$$\mathbf{A}=\begin{bmatrix}1\\0\\0\\0\\0\\0\\0\\0\end{bmatrix};\mathbf{B}=\begin{bmatrix}0\\1\\0\\0\\0\\0\\0\\0\end{bmatrix};\ \mathbf{C}=\begin{bmatrix}0\\0\\1\\0\\0\\0\\0\\0\end{bmatrix};\ \mathbf{C}'=\begin{bmatrix}0\\0\\1\\0\\0\\0\\1\\0\end{bmatrix};\mathbf{D}=\begin{bmatrix}0\\0\\0\\1\\0\\0\\0\\0\end{bmatrix};\ \mathbf{D}'=\begin{bmatrix}0\\0\\0\\1\\0\\0\\0\\1\end{bmatrix};\mathbf{E}=\begin{bmatrix}0\\0\\0\\0\\1\\0\\0\\0\end{bmatrix};\ \mathbf{F}=\begin{bmatrix}0\\0\\0\\0\\0\\1\\0\\0\end{bmatrix},$$

where C' means that the system is in stage C and the *tr-1* trigger (determining which pathway is followed by the developmental process) is on, while D' means that *tr*-2 trigger is on, which allows the system to progress toward stage E from stage D.

At the representation of fork and conditional transitions, it is more convenient to use stage vectors in which only the triggers are expressed (it is only a technical issue):

$$\mathbf{s}_{tr-1}=\mathbf{C}'-\mathbf{C}=\begin{bmatrix}0\\0\\0\\0\\0\\0\\1\\0\end{bmatrix};\ \text{and}\ \mathbf{s}_{tr-2}=\mathbf{D}'-\mathbf{D}=\begin{bmatrix}0\\0\\0\\0\\0\\0\\0\\1\end{bmatrix}.$$

The matrices of the four elementary transitions incorporated into the simple artificial differentiation hierarchy shown in Fig. 8b are the following:

- linear transition of A → B:

$$\mathbf{M}_{\mathrm{A}\to\mathrm{B}} = (2\mathbf{B}-1)\circ\mathbf{A} + (2\mathbf{B}-1)\circ\mathbf{B} = \begin{bmatrix} -1 & -1 & 0 & 0 & 0 & 0 & 0 & 0 \\ 1 & 1 & 0 & 0 & 0 & 0 & 0 & 0 \\ -1 & -1 & 0 & 0 & 0 & 0 & 0 & 0 \\ -1 & -1 & 0 & 0 & 0 & 0 & 0 & 0 \\ -1 & -1 & 0 & 0 & 0 & 0 & 0 & 0 \\ -1 & -1 & 0 & 0 & 0 & 0 & 0 & 0 \\ -1 & -1 & 0 & 0 & 0 & 0 & 0 & 0 \\ -1 & -1 & 0 & 0 & 0 & 0 & 0 & 0 \end{bmatrix},$$

- linear transition of B → C:

$$\mathbf{M}_{\mathrm{B}\to\mathrm{C}} = (2\mathbf{C}-1)\circ\mathbf{B} + (2\mathbf{C}-1)\circ\mathbf{C} = \begin{bmatrix} 0 & -1 & -1 & 0 & 0 & 0 & 0 & 0 \\ 0 & -1 & -1 & 0 & 0 & 0 & 0 & 0 \\ 0 & 1 & 1 & 0 & 0 & 0 & 0 & 0 \\ 0 & -1 & -1 & 0 & 0 & 0 & 0 & 0 \\ 0 & -1 & -1 & 0 & 0 & 0 & 0 & 0 \\ 0 & -1 & -1 & 0 & 0 & 0 & 0 & 0 \\ 0 & -1 & -1 & 0 & 0 & 0 & 0 & 0 \\ 0 & -1 & -1 & 0 & 0 & 0 & 0 & 0 \end{bmatrix},$$

- fork transition of C $\rightrightarrows$ D, F (triggered by *tr-1*):

$$\mathbf{M}_{\mathrm{C}\rightrightarrows\mathrm{D,F}}^{tr-1} = (2\mathbf{D}-\mathbf{1})\circ\mathbf{C} + 2(\mathbf{F}-\mathbf{D})\circ\mathbf{s}_{tr-1} + (2\mathbf{D}-\mathbf{1})\circ\mathbf{D} + (2\mathbf{F}-\mathbf{1})\circ\mathbf{F} =$$

$$= \begin{bmatrix} 0 & 0 & -1 & -1 & 0 & -1 & 0 & 0 \\ 0 & 0 & -1 & -1 & 0 & -1 & 0 & 0 \\ 0 & 0 & -1 & -1 & 0 & -1 & 0 & 0 \\ 0 & 0 & 1 & 1 & 0 & -1 & -2 & 0 \\ 0 & 0 & -1 & -1 & 0 & -1 & 0 & 0 \\ 0 & 0 & -1 & -1 & 0 & 1 & 2 & 0 \\ 0 & 0 & -1 & -1 & 0 & -1 & 0 & 0 \\ 0 & 0 & -1 & -1 & 0 & -1 & 0 & 0 \end{bmatrix},$$

- conditional transition of D $\nrightarrow$ E (triggered by *tr-2*):

$$\mathbf{M}_{\mathrm{D}\nrightarrow\mathrm{E}}^{tr-2} = 2(\mathbf{E}-\mathbf{D})\circ\mathbf{s}_{tr-2} + (2\mathbf{E}-\mathbf{1})\circ\mathbf{E} = \begin{bmatrix} 0 & 0 & 0 & 0 & -1 & 0 & 0 & 0 \\ 0 & 0 & 0 & 0 & -1 & 0 & 0 & 0 \\ 0 & 0 & 0 & 0 & -1 & 0 & 0 & 0 \\ 0 & 0 & 0 & 0 & -1 & 0 & 0 & -2 \\ 0 & 0 & 0 & 0 & 1 & 0 & 0 & 2 \\ 0 & 0 & 0 & 0 & -1 & 0 & 0 & 0 \\ 0 & 0 & 0 & 0 & -1 & 0 & 0 & 0 \\ 0 & 0 & 0 & 0 & -1 & 0 & 0 & 0 \end{bmatrix}.$$

The regulatory program matrix of the whole system results from the summation of the above four matrices, which reads:

$$\mathbf{M} = \begin{bmatrix} -1 & -2 & -2 & -1 & -1 & -1 & 0 & 0 \\ 1 & 0 & -2 & -1 & -1 & -1 & 0 & 0 \\ -1 & 0 & 0 & -1 & -1 & -1 & 0 & 0 \\ -1 & -2 & 0 & 1 & -1 & -1 & -2 & -2 \\ -1 & -2 & -2 & -1 & 1 & -1 & 0 & 2 \\ -1 & -2 & -2 & -1 & -1 & 1 & 2 & 0 \\ -1 & -2 & -2 & -1 & -1 & -1 & 0 & 0 \\ -1 & -2 & -2 & -1 & -1 & -1 & 0 & 0 \end{bmatrix},$$

as it is shown in the rightmost panel of Fig. 8b.

This matrix serves as the input for the dynamics described in Eq. 1. In our simplified system, the dynamics started from the developmental stage vector $\mathbf{A}$ (i.e. $\mathbf{p}(0) = \mathbf{A}$). During the integration of the system, the Pearson correlations between the actual expression vector ($\mathbf{p}(t)$) and all developmental stage vectors ($\mathbf{A}, \mathbf{B}, \ldots, \mathbf{F}$) were evaluated. We use the Pearson correlation coefficient (r) in this comparison, as it characterizes the similarity between the actual and the desired expression patterns. As the system progresses along the developmental pathway, the correlation between $\mathbf{p}(t)$ and the current stage-specific vector becomes 1 and then decreases, cf. Fig. 8b.

Supplementary Note 2. Landscape mapping

Here, we suggest an interpretation of the results of the AGRN model in the light of Waddington's epigenetic landscape view[1,2]. As such, we show that dynamical properties of the differentiation processes modeled by the AGRN framework are consistent with the image of an epigenetic landscape, where the valleys are separated by ridges, constituting barriers between stable states that can be crossed by the dynamical trajectory of the system by means of utilizing external trigger effects. This is demonstrated by a trigger perturbation analysis of the hematopoietic model (detailed in Fig. 2), indicating that some of the differentiation stages can be characterized by stronger attractor properties relative to others in terms of their frequency of being the stage into which the system-level gene expression pattern (the $\mathbf{p}(t)$ expression vector) first converges in response to a trigger (Fig. S1). Because different types of genes (triggers, unique and non-unique genes) have different effects on the behavior of the system, we analyzed the effect of perturbation of different types of genes separately.

Since triggers are the main drivers of cell-fate development, we first analyzed the effect of mistimed triggers at different stages. We allowed the system to approach each developmental stage during its normal evolution, then each trigger in the model was injected with different strength (from 0.5 to 5.0 by 0.5) and we monitored into which stage the dynamical trajectory of the system converged first with a 0.95 Pearson correlation coefficient, see Fig. S1a. Here we considered only those cases, where the next stage into which the system converged was not the perturbed stage itself or its default successor. We have also investigated the definitive power of the unique genes by switching these genes on one-by-one, while making a full-perturbation mapping of all the other genes and recorded the first fully expressed stage (Fig. S1b). To measure the effect of non-unique genes, we set triggers and stage-specific genes to 0 and initialized the simulation from the full-perturbation map of the rest of the genes either turned on or off. Then the relative frequencies of the succeeding expressed stages were measured, see Fig. S1c.

(In all simulations, we have used 0.0 and 5.0 expression levels for turning off and on the genes, respectively.)

We found that the definitive endothelial cell stage has the strongest attractor property as this is the most frequent trigger-induced differentiation stage (Fig. S1a) and this is the third most frequent and the most frequent stage in the unique and non-unique gene-mapping, respectively (Figs. S1b, c). These results suggest that the stage with the largest basin in the epigenetic landscape of hematopoiesis corresponds to an end-differentiated cellular stage.

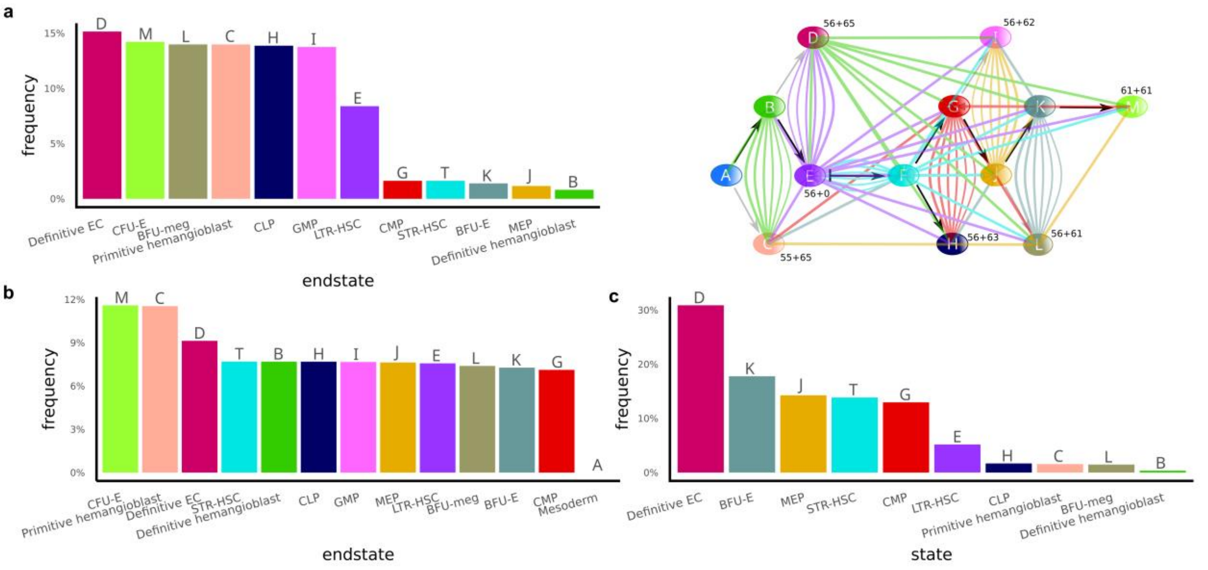

Fig. S1 Mapping of attractor pool sizes in the hematopoiesis model. a Attractor pool sizes of different stages. Colors of the arrows on the graph indicate the identity of the triggers and correspond to the color of the original target stages of individual triggers. Widths of the arrows indicate the strength of particular triggers. Ineffective triggers (where the next stage to which the system converged was the perturbed stage itself or its default successor) have been removed from the graph for clarity. Numbers next to the graph nodes represent the number of arrows removed (self-directed and default). **b** Definitive power of the unique genes. **c** Attractor pool sizes of different stages without unique genes.

Supplementary Note 3. Effect of degradation rate

We have analyzed the effect of different degradation rates (δ) on the dynamics. In line with the expectations, increasing δ decreases the characteristic times of transitions (T), which is the time difference between two consecutive 95% percent expression levels. Fig. S2 shows T as the function of the degradation rates in case of 15 consecutive heteroassociative transitions. The resulting curve is well approximated by the function $T \sim \delta^{-1}$ i.e., the characteristic time scales with the average lifetime of the gene products.

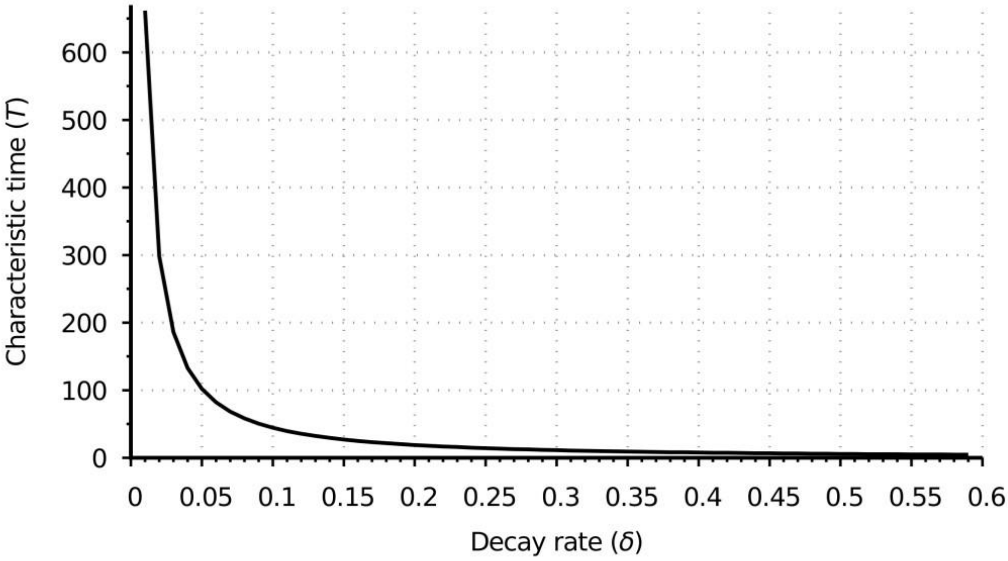

Fig. S2 The characteristic time of linear transition as the function of degradation rate (δ). T was averaged over 15 consecutive heteroassociative transitions. Parameters, except δ, are from the standard parameter set.

If the degradation rate is the same for all gene products, the system spends the same time in all stages (except in case of conditional transition, where the trigger defines this time). By introducing different degradation rates for N different gene products, which results in a decay vector $\boldsymbol{\delta}_i$ $(i = 1, \ldots, N)$, the time that the system spends in a given stage can be modified. In case of the human cell cycle model system (see Fig. 3), the appropriate product-specific degradation rates can be determined by an evolutionary algorithm. If the relative lengths of the stages are the following: $\tau_{\mathrm{G_1}} = 0.45, \tau_{\mathrm{S}} = 0.3, \tau_{\mathrm{G_2}} = 0.2\,, \tau_{\mathrm{M}} = 0.05$ (in arbitrary time units), then the algorithm results in a possible appropriate degradation rate vector:

$\boldsymbol{\delta} = (0.19, e, 0.46, 0.14, 0, 0.13, 0.16, g, g, g, f, d, c, c, c, c, b, g, c, c, f, f, c, c, e, f, c, c, b, c, f, d, b, c, c, e, c, c, b, a, a, c, c, c, f, c, d, a, c, c, f, c, a, c, a, 0)$,

where $a = 0.23$, $b = 0.24$, $c = 0.25$, $d = 0.26$, $e = 0.29$, $f = 0.3$, $g = 0.31$. (The respective order of the corresponding gene products is given at Supplementary Data 2.)

Supplementary Note 4. Regulatory network modularity

Gene regulatory network reconstruction of hematopoietic cellular populations revealed that various differentiation trajectories initiated from a common progenitor cell-type population are regulated by distinct cellular differentiation programs[3]. In terms of regulatory network topology, this means that a gene regulatory network that guides the differentiation of the complete hematopoietic hierarchy can be decomposed into several lineage-specific GRNs, of which some constitutes a more or less isolated network module. To incorporate this modularity of the regulation mechanism into the AGRN model, we constructed a hematopoietic model, analogous to the hematopoietic model shown in Fig. 2, driven by a

modular regulatory network (Fig. S3a). Thus, the major conceptual difference between the analogous hematopoietic model and this modular regulatory network modulated differentiation process is that stage transitions in the latter case are regulated by three different **M** regulatory program matrices, in contrast to the former default case, where all differentiation pathways are regulated by a single **M** regulatory program matrix. Performance of the modular model was tested on the same data as were used in Fig. 2 (Supplementary Data 1). The model performance is measured by Pearson correlation coefficients between the **p**(t) expression vector (vector for the actual dynamical expression state of the genes) and the developmental stage vectors. Fig. S3b shows this measure in case of two illustrated differentiation pathways, indicating that the modular model results in qualitatively similar gene expression dynamics to the analogous default model shown in Fig. 2**.**

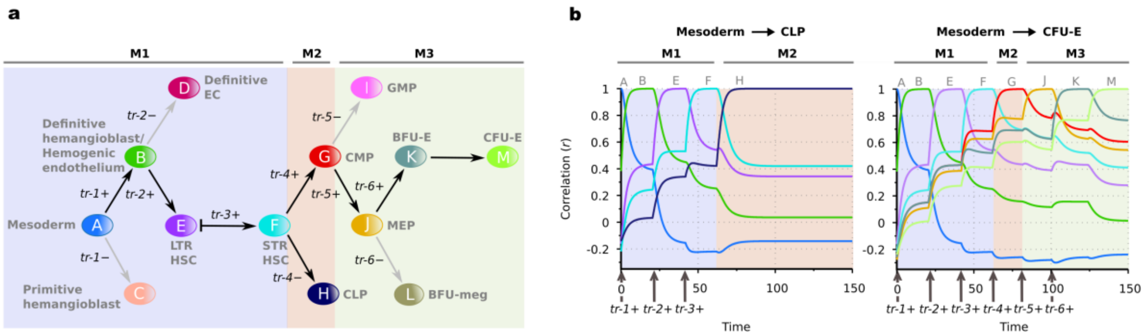

Fig. S3 Illustration of the hematopoietic cell differentiation process with a modular AGRN. Background colors denote differentiation modules regulated by different **M** regulatory program matrices. **a** Hematopoietic differentiation topology of the model. Uppercase letters with rounded and colored background represent cell differentiation stages, the arrows between them represent transitions between the stages. The fork transitions and the conditional transition are controlled by the expression of transition-specific triggers (denoted as *tr-1, tr-2, …*). The two differentiation pathways demonstrated here are highlighted with black arrows. **b** Realizations of stages, as measured by Pearson correlation coefficients between the **p**(t) expression vector and the stage-specific developmental stage vectors (Supplementary Data 1). Color code for the lines that correspond to the cellular differentiation stages is given at (**a**). Color code for the time scale of the trajectory samples with the timing of the corresponding triggers is shown on the right. For the nomenclature and topology of the differentiation hierarchy see Fig. 2.

Supplementary Note 5. An organogenetic AGRN model

Due to its invariant cell lineages[4] and the wide access to relevant gene expression data, the *Caenorhabditis elegans* vulva development is an ideal process to demonstrate that the proposed AGRN model framework is also applicable to organ development systems. Therefore, we extracted information on binary gene expression states that characterize differentiating *C. elegans* vulval cells from refs.[4–6], and constructed a dataset that consists of stage-specific gene expression profile vectors for the cellular stages of the differentiation hierarchy (Supplementary Data 4, 5). The mature *C. elegans* vulva comprises seven cell types (vulA, vulB1, vulB2, vulC, vulD, vulE, and vulF) that are descendants of the P5.p, P6.p, and P7.p vulval precursor cells (VPCs)[4]. Fig. S4 shows that starting from the P5.p, P6.p precursor cells, the model successfully reproduces the stage-specific gene expression profiles for each of the seven cell types, including the intermediate cellular differentiation stages during different larval stages. Fork transitions are controlled by the expression of a transition-specific trigger (denoted as *tr-1*, … in the P5.p, and *tr* in the P6.p hierarchy), which represents a switching mechanism that mediates

(extracellular) signals during asymmetric cell divisions. Linear transitions between adjacent stages (occurring from early L4 to adult in all cell types, except in vulD, where they start from late L3) represent a cell maturation process, where the system-level gene expression pattern switches to the next cellular stage without cell division and an external signal.

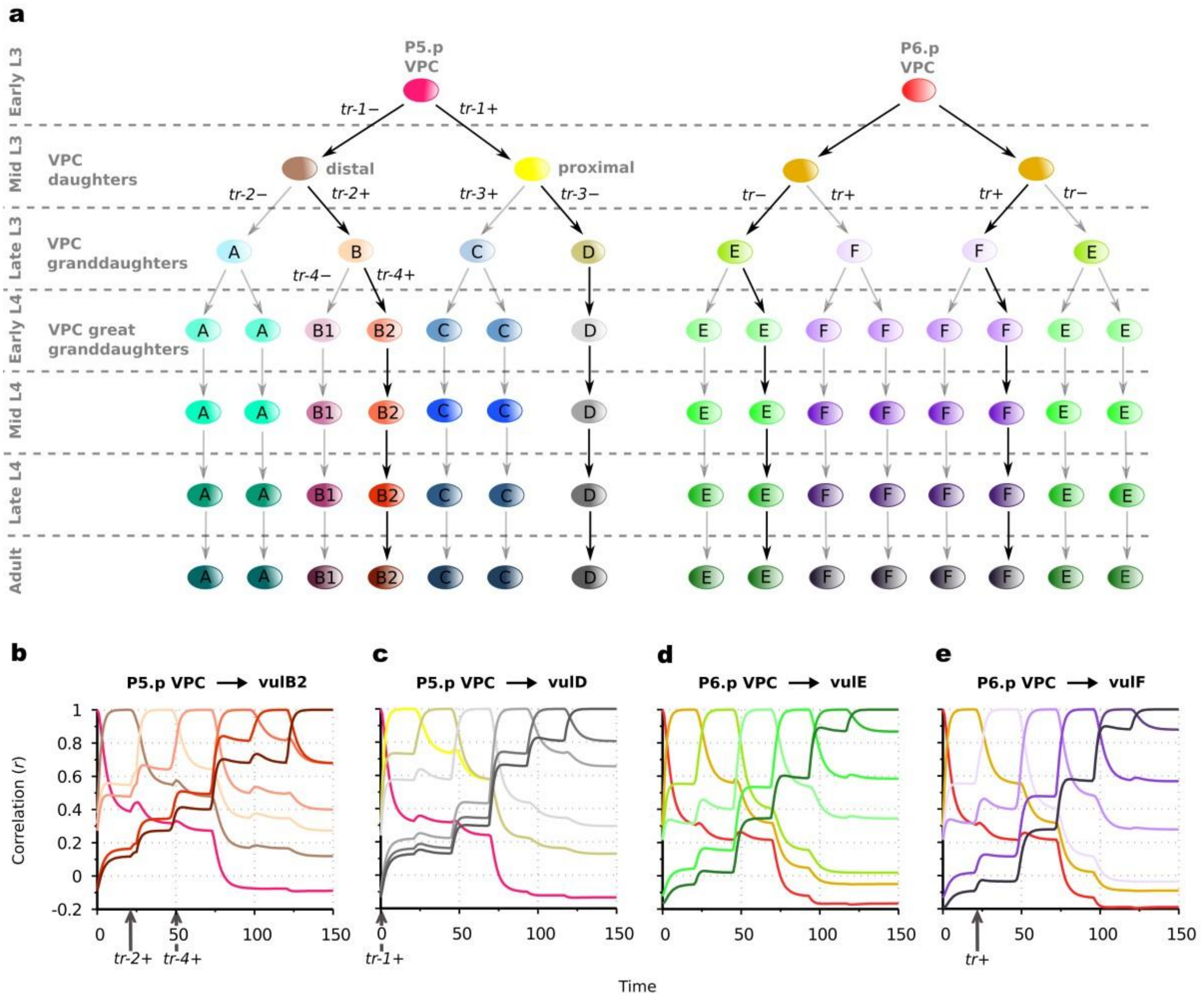

Fig. S4 Illustration of the differentiation process of P5.p and P6.p *C. elegans* vulval precursor cells (VPCs) with an associative GRN. a Differentiation topology of the model. In the nomenclature and topology of the differentiation hierarchy, we followed ref.[4]. Uppercase letters with rounded and colored background represent cell differentiation stages, the arrows between them represent transitions between the stages. Dashed lines separate organism-level developmental stages from early L3 to adult. Both in the P5.p and P6.p hierarchies, there are two illustrated differentiation pathways, each highlighted with black arrows. **b**–**e** Realizations of stages, as measured by Pearson correlation coefficients between the **p**(*t*) expression vector and the stage-specific gene expression profile vectors (Supplementary Data 4, 5). Color code for the lines that correspond to the developmental stages is given at (**a**). The P5.p VPC⟶vulB2 pathway (**b**) realizes, if two external signals – mediated by the respective triggers (*tr-2+* and *tr-4+*) – are presented at the appropriate time and order (denoted by vertical arrows at the x-axis). The P5.p VPC⟶vulD pathway (**c**) realizes, if a different type of external signal is mediated (*tr-1+*). Differentiation of P6.p VPC cell to vulE type cell (**d**) occurs in the absence of any trigger-mediated external signals. The P6.p VPC⟶vulF pathway (**e**) realizes, if an external signal is mediated by the P6.p hierarchy-specific trigger (*tr+*).

Supplementary Note 6. Subcircuits

In order to identify the network subcircuits characteristic of the regulatory program matrices of the AGRN model framework, we conducted full enumeration of three-membered motifs on the human hematopoiesis regulatory program matrix. This enumeration resulted in 5984 motifs including those that are not fully connected (there is no edge between one or more of their node-pairs). Although the analyzed matrix includes autoregulatory effects (self-directed edges), definition of different motif groups has been carried out without taking into consideration these self-directed edges for simplicity. First, we categorized the motifs into 14 main groups in which only the direction and the position of the edges between the nodes has been considered (Supplementary Table 1). Second, we constructed within-group motif categories in which the motif types were further differentiated according to the positive (green) or negative (red) regulatory effects between their nodes. This motif classification resulted in 49 within-group motif categories altogether (including the 3rd self-identical main group). Supplementary Table 1 shows these motifs listed in descending order by the frequency of their occurrences.

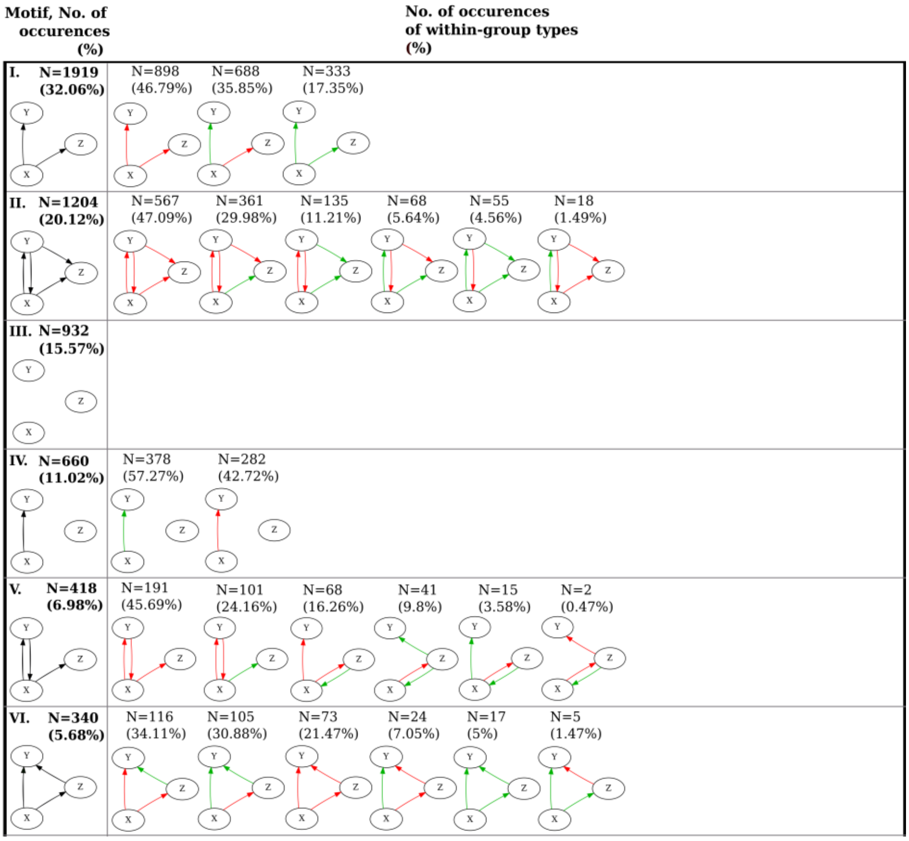

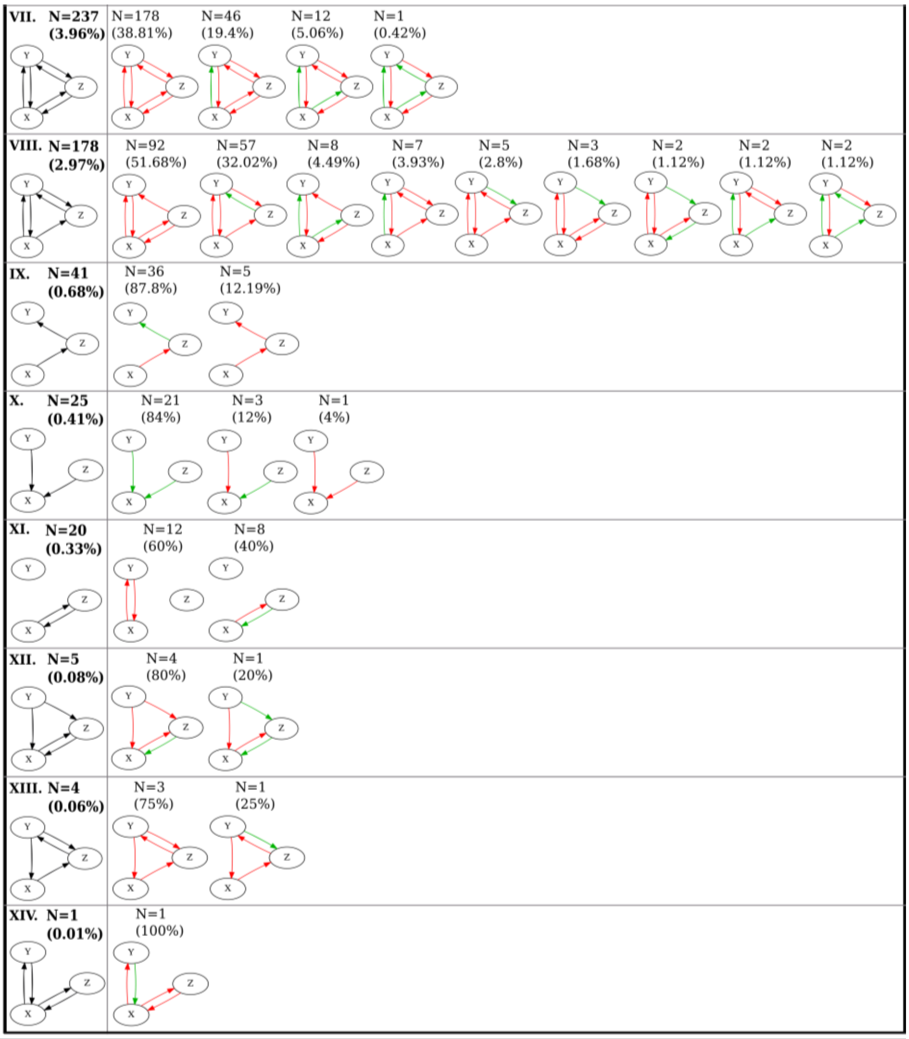

**VII. N=237 (3.96%)**	N=178 (38.81%)	N=46 (19.4%)	N=12 (5.06%)	N=1 (0.42%)					
**VIII. N=178 (2.97%)**	N=92 (51.68%)	N=57 (32.02%)	N=8 (4.49%)	N=7 (3.93%)	N=5 (2.8%)	N=3 (1.68%)	N=2 (1.12%)	N=2 (1.12%)	N=2 (1.12%)
**IX. N=41 (0.68%)**	N=36 (87.8%)	N=5 (12.19%)							
**X. N=25 (0.41%)**	N=21 (84%)	N=3 (12%)	N=1 (4%)						
**XI. N=20 (0.33%)**	N=12 (60%)	N=8 (40%)							
**XII. N=5 (0.08%)**	N=4 (80%)	N=1 (20%)							
**XIII. N=4 (0.06%)**	N=3 (75%)	N=1 (25%)							
**XIV. N=1 (0.01%)**	N=1 (100%)								

Supplementary Table 1 Three-membered subcircuits identified in the human hematopoiesis model regulatory program matrix.

Supplementary References

1. Waddington, C. H. *The Strategy of the Genes.* (London: George Allen & Unwin, 1957).
2. Enver, T., Pera, M., Peterson, C. & Andrews, P. W. Stem Cell States, Fates, and the Rules of Attraction. *Cell Stem Cell* **4**, 387–397 (2009).
3. Ainciburu, M. *et al.* Uncovering perturbations in human hematopoiesis associated with healthy aging and myeloid malignancies at single-cell resolution. *eLife* **12**, e79363 (2023).
4. Ririe, T. O., Fernandes, J. S. & Sternberg, P. W. The *Caenorhabditis elegans* vulva: A post-embryonic gene regulatory network controlling organogenesis. *Proc. Natl. Acad. Sci.* **105**, 20095–20099 (2008).
5. Inoue, T., Wang, M., Ririe, T. O., Fernandes, J. S. & Sternberg, P. W. Transcriptional network underlying *Caenorhabditis elegans* vulval development. *Proc. Natl. Acad. Sci.* **102**, 4972–4977 (2005).
6. Wagmaister, J. A., Gleason, J. E. & Eisenmann, D. M. Transcriptional upregulation of the *C. elegans* Hox gene *lin-39* during vulval cell fate specification. *Mech. Dev.* **123**, 135–150 (2006).
