# Supplementary material for: A neural network-based model framework for cell-fate decisions and development
Source: Commun Biol. 2024 Mar 14;7:323. doi: 10.1038/s42003-024-05985-1 (PMC10940658; doi:10.1038/s42003-024-05985-1)
Supplement: Supplementary file 3 — Description of Additional Supplementary Files [file 42003_2024_5985_MOESM3_ESM.pdf]

Description of Additional Supplementary Files

File name: Supplementary Data 1
Description: Developmental stage vectors containing binary gene expression profiles for the hematopoietic cell differentiation dynamics investigated in the model.

File name: Supplementary Data 2
Description: Phase-specific expression vectors containing binary gene expression profiles for the human cell-cycle dynamics investigated in the model.

File name: Supplementary Data 3
Description: Developmental stage vectors containing binary gene expression profiles for the C. elegans embryonic development dynamics investigated in the model.

File name: Supplementary Data 4
Description: Developmental stage vectors containing binary gene expression profiles for the P5.p C. elegans vulval precursor cell differentiation dynamics investigated in the model.

File name: Supplementary Data 5
Description: Developmental stage vectors containing binary gene expression profiles for the P6.p C. elegans vulval precursor cell differentiation dynamics investigated in the model.
